# Supplementary figures and images for: Large-Sample Genome-Wide Association Study of Resistance to Retained Placenta in U.S. Holstein Cows
Source: Int J Mol Sci. 2024 May 20;25(10):5551. doi: 10.3390/ijms25105551 (PMC11122073; doi:10.3390/ijms25105551)

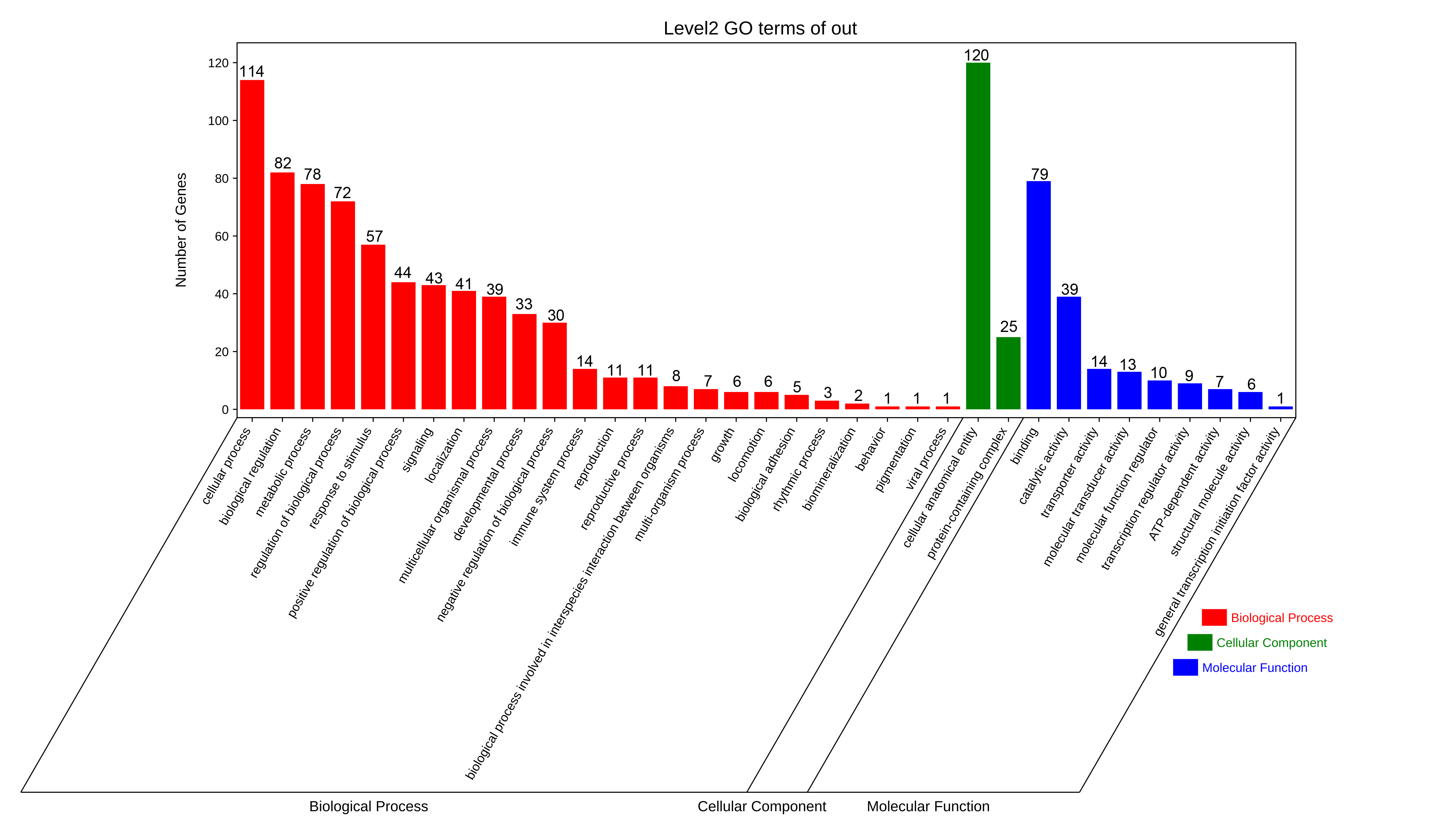

Supplement: Supplementary file 1 [file ijms-25-05551-s001.zip › Figure_S1.png]
